# Sclerostin Antibody Treatment Increases Bone Formation, Bone Mass, and Bone Strength of Intact Bones in Adult Male Rats

**DOI:** 10.1038/srep15632

**Published:** 2015-10-23

**Authors:** Pui Kit Suen, Tracy Y. Zhu, Dick Ho Kiu Chow, Le Huang, Li-Zhen Zheng, Ling Qin

**Affiliations:** 1 Musculoskeletal Research Laboratory, Department of Orthopaedics and Traumatology, The Chinese University of Hong Kong, Hong Kong SAR, PR China; 2 Bone Quality and Health Centre, Department of Orthopaedics and Traumatology, The Chinese University of Hong Kong, Hong Kong SAR, PR China; 3 The CUHK-ACC Space Medicine Centre on Health Maintenance of Musculoskeletal System, The Chinese University Research Institute in Shenzhen, PR China

## Abstract

We investigated the systemic effect of sclerostin monoclonal antibody (Scl-Ab) treatment on intact non-operated bones in an open osteotomy male Sprague Dawley (SD) rat model. Six-month-old male SD rats were subjected to transverse osteotomy at the right femur mid-shaft. Rats were injected subcutaneously with vehicle or Scl-Ab (25 mg/kg, 2 times per week) treatment for 9 weeks. Compared with vehicle control, Scl-Ab treatment significantly improved trabecular and cortical bone mass and microarchitecture at L5 vertebrae and left femora by micro-CT at week 6 and 9. Mechanical testing showed that Scl-Ab treatment resulted in significantly higher stiffness, energy to failure and ultimate load at the femora at week 9. Mineral apposition rate, mineralizing surface and bone formation rate on the trabecular bone in the distal femora was significantly increased in Scl-Ab group at week 6 and 9. The administered Scl-Ab was localized in the osteocytes and beta-catenin was strongly expressed in osteoblasts. Scl-Ab treatment significantly increased serum P1NP level and there was no between-group difference in serum level of CTX-1. In conclusion, Scl-Ab treatment could induce rapid and sustained increase in bone formation, bone mass and bone strength in non-operated bones. Sclerostin inhibition might be advantageous to prevent secondary fracture(s).

Sclerostin is a glycoprotein expressed by osteocytes as a potent regulator of bone formation. Sclerostin impedes osteoblast proliferation and function by inhibiting the Wnt/beta-Catenin signaling pathways and hence inhibits bone formation. Serum sclerostin level is evaluated with increasing age^1^. Monoclonal antibodies against sclerostin (Sclerostin monoclonal antibody, Scl-Ab) have been shown to enhance bone formation in several animal models, such as ovariectomized (OVX) rat model for simulating postmenopausal osteoporosis^2^, gonad-intact aged male rats^3^, in hindlimb immobilized rats^4^ or mice model^5^, and in gonad-intact female cynomolgus monkeys^6^. In clinical trials, Scl-Ab (Romosozumab) has been shown to increase bone mineral density (BMD) in both healthy men and postmenopausal women with low BMD^7^,^8^. Given its pivotal role in regulating bone formation, sclerostin is a promising pharmacologic target for prevention and treatment of osteoporosis.

Several studies have demonstrated positive effect of inhibition of sclerostin in fracture healing in femoral osteotomy (open fracture) models in mice^9^ and rat^10^, in closed femoral fracture model in rats^11^, and in a fibular osteotomy model in male cynomolgus monkeys^11^. In these studies, Scl-Ab has shown to significantly increase bone mass at the fracture site as well as the strength of the fracture union. Fracture begets future fracture(s). Two meta-analyses have shown a doubling of future fracture risk in patients who experience a prior fracture at any skeletal site^12^,^13^. Therapies that increase bone strength throughout the skeleton while enhancing fracture healing will have the potential to reduce the risk of a secondary fracture.

We have previously reported that Scl-Ab enhanced fracture healing in an open femoral osteotomy model in male Sprague Dawley (SD) rats by enhancing bone volume and mineralization, angiogenesis and mechanical properties^14^. In this study, we reported the effect of Scl-Ab on the non-fracture bones in this open osteotomy rat model. Bone mass, microarchitecture of trabecular bone, bone strength, dynamics of bone formation, and bone turnover markers were systemically assessed to study the anabolic effect of Scl-Ab on the intact non-operated bone.

## Results

### Micro-CT analysis of the L5 vertebra

**Figure 1:**
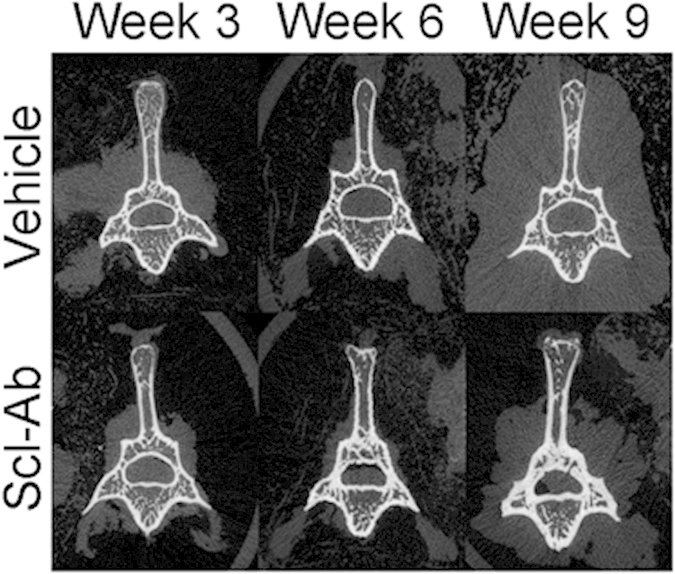
Representative micro-CT images of the L5 vertebrae of Scl-Ab treatment and the vehicle treatment. Scl-Ab treatment significantly increased cortical thickness and trabecular thickness of the L5 vertebrae.

Scl-Ab treatment improved the trabecular bone density at the 5th lumbar vertebra (L5 vertebra), with significantly higher bone volume fraction (bone volume/tissue volume, BV/TV) values at all time points and higher BMD and bone mineral content (BMC) at week 6 and 9 (Table 1). Trabecular microarchitecture was also improved with Scl-Ab treatment, with significantly increased trabecular number (Tb.N) (23%) at week 6, significantly increased trabecular thickness (Tb.Th) at all time points (25%, 75% and 90% at week 3, 6 and 9, respectively) and significantly decreased trabecular spacing (Tb.Sp) at week 6 (−24%) and 9 (−15%). At the cortical region of L5 vertebra, Scl-Ab significantly increased BMD, BMC, cross sectional area (CSA), cortical thickness (Ct.Th), cross sectional moment of inertia (CSMI), CSA derived bone strength index (BSI_CSA_) and CSMI derived bone strength index (BSI_CSMI_) at week 6 and 9 and the largest increase of these indices were observed at week 9 (10%, 87%, 69%, 54%, 86%, 87% and 105%, respectively) (Table 1). Figure 1 shows the representative micro-CT images of the L5 vertebra of Scl-Ab and vehicle treatment groups. Increase in Ct.Th and Tb.Th was significantly more prominent in the Scl-Ab treatment group (all p < 0.01 at week 9).

### Micro-CT analyses of the femora

**Figure 2:**
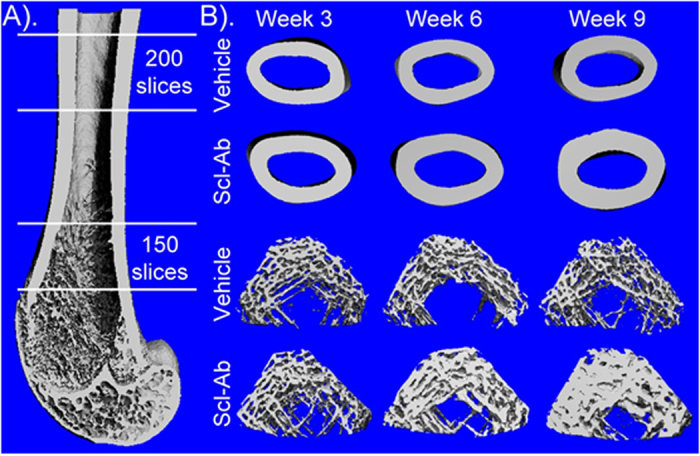
Micro-CT analysis of cortical and trabecular bone of the femora. (**A**) Region of interest (ROI) chosen for analysis including cortical compartment of the femoral midshaft and trabecular compartment of distal femoral metaphyseal. (**B**) Representative 3D micro-CT images of Scl-Ab and vehicle treated samples. Scl-Ab treatment significantly increased cortical thickness at the femoral midshaft and trabecular bone volume and thickness at the distal femoral metaphyseal at week 6 and 9.

At the trabecular region of the distal femora, Scl-Ab treatment significantly increased BV/TV, BMD and BMC at week 6 and 9 compared with the vehicle treatment with the largest increase observed for BV/TV at week 9 (333%) (Table 2). Tb.Th increased significantly at all time points while Tb.N increased and Tb.Sp decreased significantly at week 6 and 9. At the cortical region of femoral midshaft, Scl-Ab significantly increased BMC, CSA, Ct.Th, BSI_CSA_ and BSI_CSMI_ at week 6 and 9 compared with the vehicle treatment with the largest increase observed for Ct.Th at week 9 (22.9%). CSMI also increased significantly with Scl-Ab treatment at week 9 (22.7%). Cortical BMD of femoral midshaft did not change significantly with Scl-Ab treatment. Figure 2 shows the representative micro-CT images of the distal and femoral midshaft of Scl-Ab and the vehicle treatment groups. Increase in Ct.Th and Tb.Th was significantly more prominent in the Scl-Ab treatment group at week 6 and 9 (all p < 0.01).

### Mechanical properties of femur

**Figure 3:**
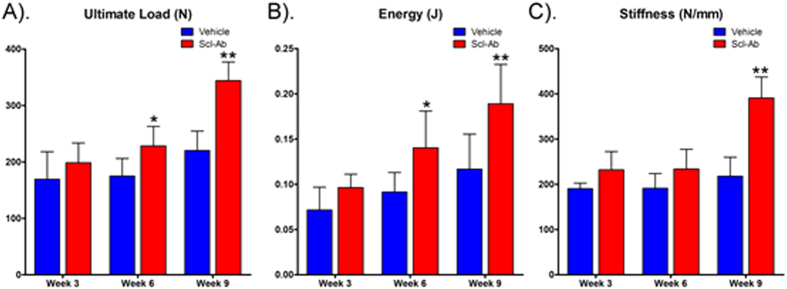
Mechanical test of the femora. Three-point bending test showed that Scl-Ab treatment significantly increased ultimate load (**A**) and energy to failure (**B**) at week 6, and significantly increased stiffness (**C**) at week 9 compared with the vehicle group at the same time point. Bars represent Mean ± SD. *P < 0.05; **P < 0.01 compared with vehicle at the same time point.

Three point bending test on the left femur showed that Scl-Ab treatment resulted in significant increase in ultimate load (31% and 56% at week 6 and 9, respectively, all p < 0.05) and energy to failure (53% and 62% at week 6 and 9, respectively, all p < 0.05) at the femora as compared with vehicle treatment at week 6 and 9 after treatment (Fig. 3). Increase in stiffness in the Scl-Ab treatment group was not significant at week 3 and 6 but was significant at week 9 (80%, p < 0.01) compared with the vehicle treatment.

### Immunohistochemistry of beta-catenin

**Figure 4:**
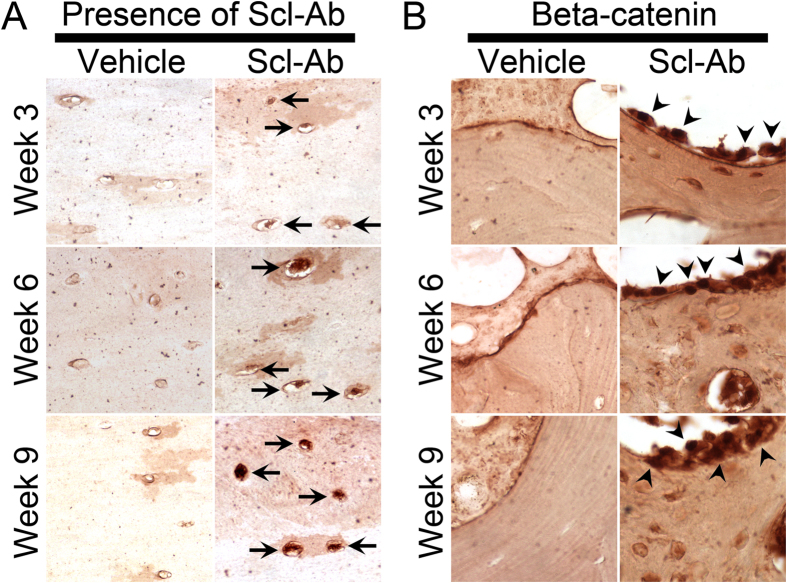
Detection of the administered Scl-Ab and immunohistochemistry of beta-catenin. (**A**) The administered Scl-Ab was concentrated in the osteocytes (arrows). Magnification: 400x. (**B**) Scl-Ab treatment induced the expression of beta-catenin in osteoblasts (arrowheads). Magnification: 400x.

To investigate whether Scl-Ab treatment induced the expression of beta-catenin, we first detected the presence of Scl-Ab at the femora. Immunohistochemistry staining using anti-human IgG antibody showed that the administered Scl-Ab was localized in the osteocytes (Fig. 4A) while vehicle treatment showed no positive staining. We then detected the beta-catenin expression by using anti-beta-catenin antibody. Staining showed that beta-catenin was strongly expressed in osteoblasts on the surface of trabecular bone (Fig. 4B).

### Histomorphometric analysis

**Figure 5:**
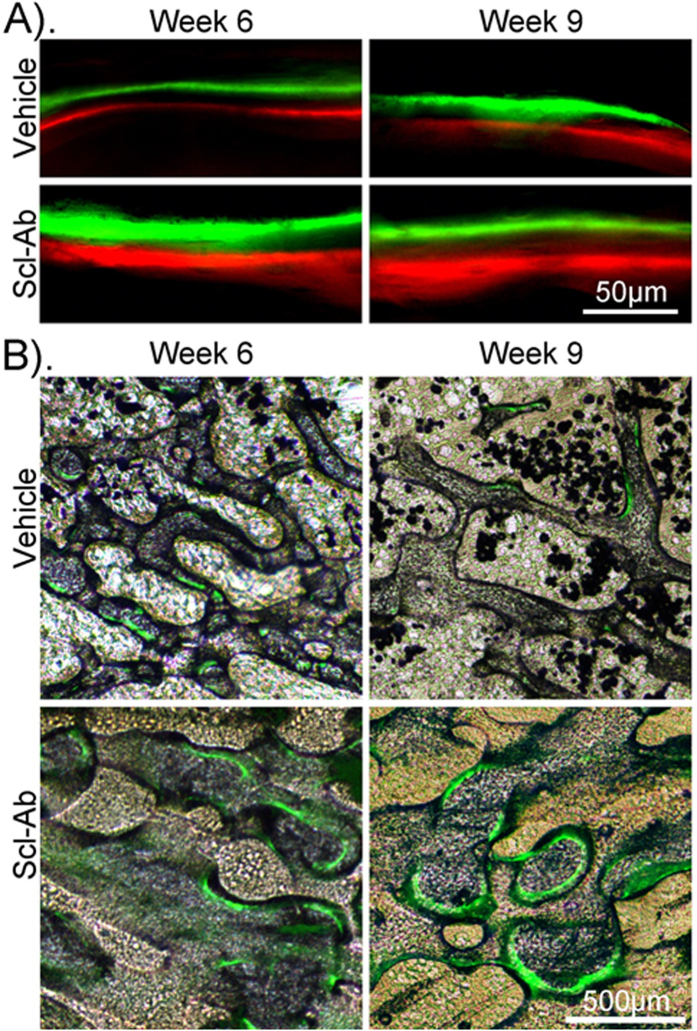
Bone formation assessed by fluorescent labeling. Scl-Ab treatment significantly enhanced new bone formation in the metaphyseal trabecular bone region of the distal femora (**A**) and increased the mineralizing surfaces (**B**). Magnification: 200x (**A**) and 25x (**B**).

Dynamic histomorphometric analysis at week 6 and 9 showed that the Scl-Ab treatment significantly enhanced bone formation. Scl-Ab treatment significantly increased mineral apposition rate (MAR) (93% and 102% at week 6 and 9, respectively), mineralizing surface (MS/BS) (270%, 433%) and bone formation rate (BFR) (592%, 977%) compared with the vehicle treatment at week 6 and 9 (Table 3). Figure 5 shows the enhanced bone mineralization after Scl-Ab treatment by sequential fluorescent labeling.

### Serum levels of bone turnover markers

Serum levels of bone formation marker procollagen type 1 N-terminal propeptide (P1NP) and bone resorption marker C-telopeptide of type 1 collagen (CTX-1) were measured. Consistent with the increased BMD in micro-CT analysis, Scl-Ab treatment significantly increased serum level of P1NP at all time points, with the largest increase observed at week 6. There was no significant difference in serum level of CTX-1 between Scl-Ab and vehicle treatment (Table 4).

## Discussion

In this study, we evaluated the effectiveness of sclerostin inhibition on bone mass, bone microarchitecture, bone strength and bone formation in the intact bones of an open osteotomy rat model. We found that Scl-Ab for up to 9-week treatment improved bone density and microarchitecture of both trabecular and cortical bone at the L5 vertebra and femur. Indices of bone formation were markedly elevated in trabecular bone. These changes were accompanied by with increased serum bone formation marker and elevated expression of beta-catenin in osteoblasts. As a result, bone strength increased substantially with Scl-Ab treatment. Our results confirmed that sclerostin is a key negative regulator of bone formation and systemic administration of Scl-Ab could promote bone formation, increase bone mass and bone strength throughout the skeleton in an open osteotomy rat model. These results support the potential of Scl-Ab as a pharmacological or biological strategy for secondary fracture prevention.

It is well recognized that fracture begets future fracture(s). Patients who suffer an initial fracture are at a greater risk of subsequent fracture(s). Therefore, an anabolic treatment that can increase bone strength throughout the skeleton while improving fracture healing should have the potential to reduce the risk of a secondary fracture^12^,^13^. We have previously reported the anabolic effect of Scl-Ab on improving fracture healing in this open osteotomy rat model^14^. In this study, the systemic Scl-Ab administration has anabolic effect that increased bone mass in the non-operated bones. These results indicate that sclerostin inhibition might be advantageous to prevent a secondary osteoporotic fracture. Sclerostin inhibition has been reported to increase BMD, BV/TV, trabecular microarchitecture and Ct.Th at multiple skeletal sites including lumbar vertebrae, femora, and tibiae in 19-month-old OVX rats, 10-month-old intact female rats and gonad-intact female cynomolgus monkeys^2^,^4^,^6^. Our study further demonstrated that 9-week Scl-Ab treatment not only increased bone mass, improved trabecular microarchitecture and Ct.Th, but also improved geometric parameters (CSA, CSMI, BSI) of the cortical bone at the L5 vertebra and femur in a young orthopaedic rat model. Some of the improvement, such as increases in Tb.Th at the L5 vertebra and distal femur, were already evident at week 3, while most of the significant improvement occurred at week 6 and was maintained at week 9, indicating a sustained effect of Scl-Ab on the bones. It is well known that deterioration of trabecular and cortical microarchitecture is a risk factor for reduced bone strength and heightened fracture in human^15^,^16^. We have previously shown that the two BSIs incorporating both material and geometric properties are highly correlated with mechanical properties of the bone^17^. The marked improvement in density and structure by Scl-Ab treatment translated into significant increases in the ultimate bone strength as demonstrated by mechanical testing.

In addition, significant improvements of trabecular BMC were observed in Scl-Ab treatment group on both L5 vertebra and distal femur. These improvements were likely the results of increased bone volume with Scl-Ab treatment, as reflected by increased trabecular BV/TV, as well as increased Tb.N and Tb.Th. Similar anabolic effects are also observed in cortical BMC, thickness and area of both L5 vertebra and femur midshaft. These findings are also consistent with previous studies^2^,^3^. In contrast, increases in trabecular and cortical BMD appeared to be less compared with those in BMC. This may be possibly due to the fact that the rats were reaching their peak bone mass and bone quality was improved through improving bone architecture instead of bone density. The effects of Scl-Ab on bone turnover markers were also investigated. We showed significant anabolic effect of Scl-Ab treatment through increased bone formation without increased bone resorption marker, thus uncoupled bone turnover. Similar anabolic effects of Scl-Ab treatment were also observed in previous studies in a rat closed femoral fracture model^11^, non-fractured aged male rats^3^ and in gonad-intact female cynomolgus monkeys^6^.

Sclerostin antagonizes Wnt/beta-catenin signaling pathway partly by binding to the extracellular domain of low-density lipoprotein receptor-related protein 5/6 and thereby decreases bone formation^18^,^19^. We showed that the administered Scl-Ab was localized in the osteocytes, the major cell type that expresses sclerostin. Sclerostin inhibition by Scl-Ab led to increased expression of beta-catenin in osteoblasts, increasing bone formation. In fact that Sclerostin inhibition has shown to markedly increase bone formation in several animal models^2^,^3^,^4^,^6^. Consistent with previous findings, we showed that Scl-Ab treatment improved the MAR, MS/BS and BFR in the distal femur, suggesting a significant increase in the rate of mineralized bone deposition and an increase in osteoblast recruitment and functional longevity. Our findings strongly supported that sclerostin is an pivotal negative regulator of bone formation and Scl-Ab treatment can markedly promote bone formation and increase bone mass, improve bone microarchitecture and improve bone strength.

Our finding that Scl-Ab treatment significantly increased bone formation by using histomorphometric analysis is consistent with previous observations on the anabolic effect of Scl-Ab in human by using bone turnover markers. In two randomized placebo-controlled clinical trials, Scl-Ab treatment have been shown to markedly increase bone formation markers while decreasing bone resorption markers in healthy men or postmenopausal women^8^ and in postmenopausal women with low bone mass^7^. These changes of bone turnover markers corresponded to a significant increase in BMD of the lumbar spine and hip using Dual-energy X-ray Absorptiometry. Together with our study, these results provided evidence that sclerostin is an emerging therapeutic target for the prevention and treatment of osteoporosis in men and postmenopausal women.

There were several limitations of our study. Firstly, we did not perform mechanical compression test on the vertebrae to determine the change of mechanical properties. Secondly, the dose-response curve of Scl-Ab in bone formation was not determined in this study. Future clinical studies are need to further explore the potential utility of Scl-Ab in the prevention of secondary fracture in human.

In conclusion, in a young open osteotomy rat model, Scl-Ab treatment could induce rapid and sustained increase in bone formation, bone mass and bone strength in non-operated bones. Our results confirmed that sclerostin is a pivotal negative regulator of bone formation and suggest that sclerostin inhibition might be advantageous to prevent a secondary osteoporotic fracture.

## Methods

### Animals and treatment

As previously described^14^, a total of 120 six-month-old male SD rats were obtained from the Laboratory Animal Services Center of the Chinese University of Hong Kong. All experimental protocol were approved by the Animal Experimentation Ethics Committee of the Chinese University of Hong Kong (AEEC No. 12/020/MIS). All animal experiments were performed in accordance with ARRIVE (Animal Research: Reporting of *In Vivo* Experiments) guidelines (National Centre for the Replacement, Refinement and Reduction of Animals in Research, UK)^20^. Osteotomy was performed under general anesthesia at mid-shaft of the right femur using a circular saw with a diameter of 1.6 cm and a thickness of 0.1 mm (Fine Science Tools, Foster City, CA) and stabilized by intramedullary insertion of a sterilized 1.2 mm diameter Kirschner wire (Stryker China, Hong Kong, China). Rats were randomly assigned to Scl-Ab treatment group (Scl-Ab IV, subcutaneous injection, 25 mg/kg, 2 times per week) or vehicle (saline) treatment group for up to 9 weeks. A total of 84 rats (42 from each group) were included for this study. At week 3, 6 and 9, 14 rats from each treatment group were terminated by administration of overdosed pentobarbital. The 5^th^ lumbar (L5) vertebrae and left femora were collected and subjected to micro-CT scan. After micro-CT scan, the femora were subjected to mechanical testing (n = 8, for each group and each time point), immunohistochemistry analysis (n = 6, for each group and each time point), and dynamic histomorphometric analysis (n = 6, for each group and time point). Sera (n = 6, for each group and time point) were collected for analysis of bone turnover markers.

### Antibody

The sclerostin monoclonal antibody was provided by Amgen (Amgen Inc., Thousand Oak, CA, US). The same compound has been commonly used in previous studies and showed strong bone anabolic effects, with dose-dependent increases on both trabecular and cortical bone formation^3^,^6^. It has a half-life in serum of approximately 14 days at a dose of 30 mg/kg body weight^6^. The dose (25 mg/kg) used in this study was based on previous study using male SD rats^3^.

### Micro-CT assessment

The L5 vertebrae and left femora were scanned by a micro-CT system (μCT-40, Scanco Medical, Brüttisellen, Switzerland). The 3D reconstruction of the mineralized tissue was performed as described previously^14^,^21^,^22^. The resolution was 30 μm and 19 μm for L5 vertebrae and femora, respectively. The trabecular bone region of the vertebrae and distal femora were chosen for analysis of the microarchitectural parameters, including trabecular thickness (Tb.Th), trabecular number (Tb.N) and trabecular spacing (Tb.Sp), bone volume fraction (BV/TV), bone mineral content (BMC) and bone mineral density (BMD). For the vertebrae, 200 slices covering the whole L5 vertebra were used for analysis. For distal femora, the metaphyseal trabecular bone containing 150 slices above the most for proximal portion of epiphyseal line was evaluated. The cortical region of the vertebra and femoral midshaft were analyzed for BMC and BMD. The geometric parameters, including cortical thickness (Ct.Th), cross section area (CSA), cross-section moment of inertia (CSMI), and bone strength indices (BSI_CSA_ = BMD × CSA and BSI_CSMI_ = BMD × CSMI)^17^, were also analyzed.

### Mechanical test

A 3-point bending test was performed on the left femora as described previously^23^ using a material testing machine (H25KS Hounsfield Test Equipment Ltd. Redhill, Surrey, UK). The femora were placed with anterior surface facing up, centered on the supports at 26 mm apart. Load was applied at a rate of 5 mm/min until failure. Ultimate load (N), energy (J) and stiffness (N/mm) were calculated from the load–deformation curve using the built-in software (QMAT Professional Material testing software, Hounsfield Test Equipment Ltd. Redhill, Surrey, UK).

### Immunohistochemical staining

Immunohistochemical staining was performed to analyze beta-catenin expression and presence of administered humanized Scl-Ab. The phosphate buffered formalin was used to fixe femora that were then decalcified by 5% formic acid for two weeks, embedded in paraffin and sectioned into five-micrometer thickness. After removal of paraffin and rehydration, sections were quenched with 0.5% hydrogen peroxide for 20 min, and treated with 10 mM citric acid for 10 min at 65 °C for antigen retrieval. Blocking was performed using 5%BSA in 1x PBS for 1 hr at room temperature. The sections were incubated with either rabbit monoclonal anti-beta-cetenin antibody (1:200, Abcam, Cambridge, MA, US) or rabbit anti-Human IgG antibody (1:200, Abcam), in a humid chamber for overnight at 4 °C. These sections were then incubated with horseradish peroxidase conjugated anti-rabbit IgG antibody (Abcam) at room temperature for 30 min and developed using DAB (Thermo Scientific, Fremont, CA, US). These sections were evaluated under light microscope (Zeiss Aixoplan with Spot RT digital camera, Zeiss, German).

### Dynamic histomorphometric analysis

Dynamic histomorphometric analysis was performed on week 6 and 9 samples. Sequential fluorescent labeling was used to study the dynamics of bone formation as described in our established protocol^14^,^21^,^22^, with calcein green and xylenol orange (10 mg/kg and 90 mg/kg, respectively, Sigma Aldrich, USA) subcutaneously injected 2 week and 1 week, respectively, before euthanasia. The femora were embedded in methyl methacrylate (MMA) without decalcification, sectioned, ground and polished to 100 μm and observed using a fluorescence microscope (Leica DM5500, Leica, Germany). The mineralizing surface (MS/BS), mineral apposition rate (MAR), and bone formation rate (BFR) of the distal femoral metaphysis were measured by OsteoMeasure (OsteoMetrics Inc., Decatur, GA, USA).

### Serum levels of bone turnover markers

The serum concentration of bone formation marker procollagen type 1 N-terminal propeptide (P1NP) and bone resorption marker C-telopeptide of type 1 collagen (CTX-1) were measured using commercially available ELISA kits (GenAsia, Shanghai, China).

### Statistical analysis

All data were expressed as mean ± SD. Two-way ANOVA with Bonferroni *posthoc* test was used to compare the differences between the Scl-Ab treatment group and the vehicle group across time points. P value < 0.05 was considered significant. All analyses were performed using GraphPad Prism 5 (California, USA).

### Ethics approval

The study was conducted with the approval from the Animal Experimentation Ethics Committee of the Chinese University of Hong Kong (AEEC No. 12/020/MIS).

## Additional Information

**How to cite this article**: Suen P. K. *et al.* Sclerostin Antibody Treatment Increases Bone Formation, Bone Mass, and Bone Strength of Intact Bones in Adult Male Rats. *Sci. Rep.* **5**, 15632; doi: 10.1038/srep15632 (2015).

## Figures and Tables

**Table 1:** Micro-CT assessment of trabecular and cortical bone of the 5^th^ lumbar vertebra.

Variables	Week 3		Week 6		Week 9	
	Vehicle	Scl-Ab	Vehicle	Scl-Ab	Vehicle	Scl-Ab
Trabecular region						
BV/TV	0.11 ± 0.01	0.16 ± 0.03**	0.10 ± 0.03	0.30 ± 0.04**	0.19 ± 0.04	0.41 ± 0.08**
BMD (mg HA/cm^3^)	741.5 ± 17.7	757.2 ± 14.5	749.7 ± 18.4	772.7 ± 20.8*	779.3 ± 17.0	815.1 ± 10.0**
BMC (mg HA)	1,461 ± 427	1,442 ± 301	1,074 ± 345	3,248 ± 505**	1,998 ± 430	4,722 ± 892**
Tb.N (1/mm)	2.61 ± 0.17	2.59 ± 0.51	2.36 ± 0.42	2.90 ± 0.32*	2.65 ± 0.36	2.85 ± 0.20
Tb.Th (mm)	0.08 ± 0.01	0.10 ± 0.01*	0.08 ± 0.01	0.14 ± 0.02**	0.10 ± 0.01	0.19 ± 0.02**
Tb.Sp (mm)	0.37 ± 0.05	0.42 ± 0.10	0.45 ± 0.09	0.34 ± 0.05**	0.40 ± 0.05	0.34 ± 0.04*
Cortical region						
BMD (mg HA/cm^3^)	715.6 ± 24.3	728.7 ± 11.6	710.9 ± 35.6	745.1 ± 31.3*	725.1 ± 41.4	798.4 ± 28.5**
BMC (mg HA)	7,854 ± 615	8,534 ± 736	7,418 ± 833	11,434 ± 1482**	7,776 ± 565	14,530 ± 1181**
CSA (mm^2^)	9.39 ± 0.80	10.01 ± 0.82	8.91 ± 0.79	13.1 ± 1.31**	9.20 ± 0.86	15.6 ± 1.18**
Ct.Th (mm)	0.24 ± 0.02	0.26 ± 0.01	0.23 ± 0.01	0.31 ± 0.02**	0.24 ± 0.02	0.37 ± 0.02**
CSMI (mm^4^)	131.4 ± 22.2	143.0 ± 26.5	129.1 ± 18.0	200.1 ± 30.0**	126.4 ± 20.6	235.1 ± 46.2**
BSI_CSA_	6,713 ± 526	7,294 ± 629	6,340 ± 712	9,772 ± 1,267**	6,647 ± 483	12,419 ± 1,009**
BSI_CSMI_	93,591 ± 13,053	104,184 ± 19,311	91,917 ± 14,718	149,435 ± 25,958**	91,358 ± 13,923	187,537 ± 36,107**

Values are mean ± SD, *P < 0.05; **P < 0.01 compared with vehicle at the same time point. Scl-Ab: sclerostin antibody; BV/TV: bone volume fraction; BMD: bone mineral density; BMC: bone mineral content; Tb.N: trabecular number; Tb.Th: trabecular thickness; Tb.Sp: trabecular spacing; CSA: cross sectional area; Ct.Th: cortical thickness; CSMI: cross sectional moment of inertia; BSI_CSA_: CSA derived bone strength index; BSI_CSMI_: CSMI derived bone strength index.

**Table 2:** Micro-CT assessment of trabecular bone region at the distal femur and cortical bone region at the femoral midshaft.

Variables	Week 3		Week 6		Week 9	
	Vehicle	Scl-Ab	Vehicle	Scl-Ab	Vehicle	Scl-Ab
Distal femur - Trabecular region						
BV/TV	0.07 ± 0.04	0.08 ± 0.02	0.07 ± 0.03	0.20 ± 0.03**	0.09 ± 0.03	0.39 ± 0.06**
BMD (mg HA/cm^3^)	708 ± 36	726.3 ± 23.4	704 ± 16	760 ± 10**	721 ± 16	804 ± 10**
BMC (mg HA)	1,677 ± 864	2,008 ± 677	1,528 ± 644	5,081 ± 1,167**	2,187 ± 657	11,461 ± 2,895**
Tb.N (1/mm)	1.07 ± 0.27	1.11 ± 0.26	1.00 ± 0.32	1.80 ± 0.30**	1.37 ± 0.28	2.16 ± 0.18**
Tb.Th (mm)	0.05 ± 0.01	0.07 ± 0.01*	0.06 ± 0.01	0.11 ± 0.01**	0.06 ± 0.01	0.18 ± 0.03**
Tb.Sp (mm)	0.88 ± 0.36	0.88 ± 0.25	1.03 ± 0.33	0.46 ± 0.09**	0.69 ± 0.16	0.28 ± 0.04*
Femoral midshaft - Cortical region						
BMD (mg HA/cm^3^)	907 ± 25	924 ± 22	921 ± 29	967 ± 19	988 ± 24	970 ± 16
BMC (mg HA)	29,583 ± 2,143	31,525 ± 2,852	30,404 ± 3,244	36,679 ± 3,095**	32,954 ± 1,675	38,839 ± 3,117**
CSA (mm^2^)	8.67 ± 0.55	9.07 ± 0.70	8.77 ± 0.86	10.07 ± 0.69**	8.86 ± 0.32	10.64 ± 0.85**
Ct.Th (mm)	0.67 ± 0.03	0.68 ± 0.05	0.67 ± 0.05	0.77 ± 0.04**	0.70 ± 0.05	0.86 ± 0.04**
CSMI (mm^4^)	33.5 ± 4.4	36.1 ± 6.1	34.3 ± 5.3	39.0 ± 6.1	32.3 ± 3.1	39.6 ± 7.0*
BSI_CSA_	7,864 ± 570	8,380 ± 758	8,082 ± 862	9,750 ± 823**	8,760 ± 445	10,324 ± 829**
BSI_CSMI_	30,368 ± 4,224	33,384 ± 6,056	31,615 ± 5,192	37,794 ± 6,328*	31,900 ± 2,960	38,053 ± 6,414*

Values are mean ± SD, *P < 0.05; **P < 0.01 compared with vehicle at the same time point. Scl-Ab: sclerostin antibody; BV/TV: bone volume fraction; BMD: bone mineral density; BMC: bone mineral content; Tb.N: trabecular number; Tb.Th: trabecular thickness; Tb.Sp: trabecular spacing; CSA: cross sectional area; Ct.Th: cortical thickness; CSMI: cross sectional moment of inertia; BSI_CSA_: CSA-derived bone strength index; BSI_CSMI_: CSMI-derived bone strength index.

**Table 3:** Histomorphometric analysis of trabecular bone in the distal femur.

Variable	Week 6		Week 9	
	Vehicle	Scl-Ab	Vehicle	Scl-Ab
MAR (μm/day)	0.57 ± 0.20	1.10 ± 0.07**	0.61 ± 0.09	1.23 ± 0.09**
MS/BS (%)	4.36 ± 1.35	16.1 ± 2.91**	4.48 ± 1.61	23.9 ± 6.59**
BFR (μm^3^/μm^2^/day)	2.59 ± 1.27	17.9 ± 4.05**	2.70 ± 0.99	29.1 ± 6.68**

Values are mean ± SD, **P < 0.01 compared with vehicle at the same time point. Scl-Ab: sclerostin antibody; MAR: mineral apposition rate; MS/BS: mineralizing surface; BFR: bone formation rate.

**Table 4:** Analysis of serum bone turnover markers.

Variables	Week 3		Week 6		Week 9	
	Vehicle	Scl-Ab	Vehicle	Scl-Ab	Vehicle	Scl-Ab
P1NP (ng/mL)	34.22 ± 9.22	48.56 ± 6.10*	33.76 ± 8.84	64.66 ± 9.40**	80.34 ± 13.32	98.44 ± 6.05*
CTX-1 (ng/mL)	1.83 ± 0.05	1.95 ± 0.12	2.03 ± 0.38	1.91 ± 0.32	2.45 ± 0.18	2.50 ± 0.27

Values are mean ± SD, *P < 0.05; **P < 0.01 compared with vehicle at the same time point. Scl-Ab: sclerostin antibody; P1NP: procollagen type 1 N-terminal propeptide. CTX-1: C-terminal telopeptide of type 1 collagen.

## References

[b1] Modder U. I. *et al.* Relation of age, gender, and bone mass to circulating sclerostin levels in women and men. J. Bone Miner. Res. 26, 373–379, 10.1002/jbmr.217 (2011). 20721932 PMC3179347

[b2] Li X. *et al.* Sclerostin antibody treatment increases bone formation, bone mass, and bone strength in a rat model of postmenopausal osteoporosis. J. Bone Miner. Res. 24, 578–588, 10.1359/jbmr.081206 (2009). 19049336

[b3] Li X. *et al.* Inhibition of sclerostin by monoclonal antibody increases bone formation, bone mass, and bone strength in aged male rats. J. Bone Miner. Res. 25, 2647–2656, 10.1002/jbmr.182 (2010). 20641040

[b4] Tian X., Jee W. S., Li X., Paszty C. & Ke H. Z. Sclerostin antibody increases bone mass by stimulating bone formation and inhibiting bone resorption in a hindlimb-immobilization rat model. Bone 48, 197–201, 10.1016/j.bone.2010.09.009 (2011). 20850580

[b5] Spatz J. M. *et al.* Sclerostin antibody inhibits skeletal deterioration due to reduced mechanical loading. J. Bone Miner. Res. 28, 865–874, 10.1002/jbmr.1807 (2013). 23109229 PMC4076162

[b6] Ominsky M. S. *et al.* Two doses of sclerostin antibody in cynomolgus monkeys increases bone formation, bone mineral density, and bone strength. J. Bone Miner. Res. 25, 948–959, 10.1002/jbmr.14 (2010). 20200929

[b7] McClung M. R. *et al.* Romosozumab in postmenopausal women with low bone mineral density. N. Engl. J. Med. 370, 412–420, 10.1056/NEJMoa1305224 (2014). 24382002

[b8] Padhi D., Jang G., Stouch B., Fang L. & Posvar E. Single-dose, placebo-controlled, randomized study of AMG 785, a sclerostin monoclonal antibody. J. Bone Miner. Res. 26, 19–26, 10.1002/jbmr.173 (2011). 20593411

[b9] Cui L. *et al.* Time-dependent effects of sclerostin antibody on a mouse fracture healing model. J Musculoskelet Neuronal Interact 13, 178–184 (2013). 23728104

[b10] Feng G., Chang-Qing Z., Yi-Min C. & Xiao-Lin L. Systemic administration of sclerostin monoclonal antibody accelerates fracture healing in the femoral osteotomy model of young rats. Int Immunopharmacol 24, 7–13, 10.1016/j.intimp.2014.11.010 (2015). 25479724

[b11] Ominsky M. S. *et al.* Inhibition of sclerostin by monoclonal antibody enhances bone healing and improves bone density and strength of nonfractured bones. J. Bone Miner. Res. 26, 1012–1021, 10.1002/jbmr.307 (2011). 21542004

[b12] Klotzbuecher C. M., Ross P. D., Landsman P. B., Abbott T. A. 3rd & Berger M. Patients with prior fractures have an increased risk of future fractures: a summary of the literature and statistical synthesis. J. Bone Miner. Res. 15, 721–739, 10.1359/jbmr.2000.15.4.721 (2000). 10780864

[b13] Kanis J. A. *et al.* A meta-analysis of previous fracture and subsequent fracture risk. Bone 35, 375–382, 10.1016/j.bone.2004.03.024 (2004). 15268886

[b14] Suen P. K. *et al.* Sclerostin monoclonal antibody enhanced bone fracture healing in an open osteotomy model in rats. J. Orthop. Res. 32, 997–1005, 10.1002/jor.22636 (2014). 24782158

[b15] Boutroy S. *et al.* Finite element analysis based on *in vivo* HR-pQCT images of the distal radius is associated with wrist fracture in postmenopausal women. J. Bone Miner. Res. 23, 392–399 (2008). 17997712 10.1359/jbmr.071108

[b16] Wachter N. J. *et al.* Correlation of bone mineral density with strength and microstructural parameters of cortical bone *in vitro*. Bone 31, 90–95 (2002). 12110418 10.1016/s8756-3282(02)00779-2

[b17] Siu W. S., Qin L. & Leung K. S. pQCT bone strength index may serve as a better predictor than bone mineral density for long bone breaking strength. J. Bone Miner. Metab. 21, 316–322, 10.1007/s00774-003-0427-5 (2003). 12928834

[b18] van Bezooijen R. L. *et al.* Sclerostin is an osteocyte-expressed negative regulator of bone formation, but not a classical BMP antagonist. J. Exp. Med. 199, 805–814, 10.1084/jem.20031454 (2004). 15024046 PMC2212719

[b19] Winkler D. G. *et al.* Osteocyte control of bone formation via sclerostin, a novel BMP antagonist. EMBO J. 22, 6267–6276, 10.1093/emboj/cdg599 (2003). 14633986 PMC291840

[b20] Kilkenny C., Browne W. J., Cuthill I. C., Emerson M. & Altman D. G. Improving bioscience research reporting: the ARRIVE guidelines for reporting animal research. PLoS Biol 8, e1000412, 10.1371/journal.pbio.1000412 (2010). 20613859 PMC2893951

[b21] Chow D. H. *et al.* Extracorporeal shockwave therapy for treatment of delayed tendon-bone insertion healing in a rabbit model: a dose-response study. Am. J. Sports Med. 40, 2862–2871, 10.1177/0363546512461596 (2012). 23075803

[b22] Zhang G. *et al.* A delivery system targeting bone formation surfaces to facilitate RNAi-based anabolic therapy. Nat. Med. 18, 307–314, 10.1038/nm.2617 (2012). 22286306

[b23] He Y. X. *et al.* Impaired bone healing pattern in mice with ovariectomy-induced osteoporosis: A drill-hole defect model. Bone 48, 1388–1400, 10.1016/j.bone.2011.03.720 (2011). 21421090

